# Knowledge Discovery on Cryptocurrency Exchange Rate Prediction Using Machine Learning Pipelines

**DOI:** 10.3390/s22051740

**Published:** 2022-02-23

**Authors:** Zeinab Shahbazi, Yung-Cheol Byun

**Affiliations:** Department of Computer Engineering, Major of Electronic Engineering, Institute of Information Science & Technology, Jeju National University, Jeju 63243, Korea; zeinab.sh@jejunu.ac.kr

**Keywords:** exchange rate prediction, cryptocurrency, XGBoost, blockchain

## Abstract

The popularity of cryptocurrency in recent years has gained a lot of attention among researchers and in academic working areas. The uncontrollable and untraceable nature of cryptocurrency offers a lot of attractions to the people in this domain. The nature of the financial market is non-linear and disordered, which makes the prediction of exchange rates a challenging and difficult task. Predicting the price of cryptocurrency is based on the previous price inflations in research. Various machine learning algorithms have been applied to predict the digital coins’ exchange rate, but in this study, we present the exchange rate of cryptocurrency based on applying the machine learning XGBoost algorithm and blockchain framework for the security and transparency of the proposed system. In this system, data mining techniques are applied for qualified data analysis. The applied machine learning algorithm is XGBoost, which performs the highest prediction output, after accuracy measurement performance. The prediction process is designed by using various filters and coefficient weights. The cross-validation method was applied for the phase of training to improve the performance of the system.

## 1. Introduction

Cryptocurrency is a type of digital asset regarding the technologies and protocols of cryptocurrency, e.g., blockchain, which runs based on a decentralized network and contains a secure platform for a transaction which reduces the records of fake processes in the network. The further explanation is related to traditional currencies, which are centralized and contain enough attractions in the ecosystem of blockchain in recent years for the highest record of exchange rate [1]. The prediction of finances is one of the challenging areas in market data, which contains the highest degree of uncertainty, relationships, and quietness. Based on the market position and innovation of digital currencies, lots of research develops the rate of the exchange prediction problem. In the recent studies [2,3], Artificial Intelligence (AI) algorithms, e.g., Artificial Neural Network (ANN), Support Vector Regression (SVR), and Bayesian Neural Network (BNN), are applied for bitcoin price prediction. The approaches from AI algorithms permit the extraction of hidden information and novel patterns from a huge amount of data, with the requirement of having any amount of knowledge regarding the dataset. The transformation of digital economics causes a significant interruption in almost all financial systems and economics, becoming fast in the digital world. Based on recent analyses and records, the digital economy size in 2025 will increase by 25% in terms of tangible and intangible digital assets [4]. The biggest problem for traders is the volatile exchange rate of digital coins. Accordingly, developing a model to clear the price of digital coins for exchanging is a meaningful process. Based on the authorship aspect of digital coins, the acceptance rate increases publicly with high records. According to this, in 2015, almost one hundred thousand companies officially agreed. Popular companies, such as Amazon, Victoria’s Secret, Gap, etc., are on this list of companies. The cryptocurrency exchange process per transaction is a record of the ledger that encompasses the public key of the users that are senders and receivers. The public key is the wallet address. The sender is supposed to set the personal key for every transaction, and after confirmation, the transaction will be broadcast in a network. A cryptocurrency network needs the confirmation of minors for transactions or exchanges, and after confirmation, the transaction is stamped as genuine all over the system. After the exchange completion, the transaction process will stop, and during this, the sender or the minor will get a reward for further exchange expenses. Based on the defined process, High-Frequency Trading (HFT) has effects on the short-period benefits, allowing traders to obtain their profits from transactions of large records [5].

The main contributions of this research are:

- Applying Extreme Gradient Boosting (XGBoost) to verify the trend classification using technical indicators for cryptocurrency;
- The exchange rate prediction is processed on Ether, Litecoin, and Monero;
- The proposed system focuses on the prediction model’s performance to improve the accuracy of the exchange rate of digital coins;
- Identifying the techniques of feature selection for related attributes.;
- Knowledge discovery from the predicted cryptocurrency exchange rate for higher system performance;
- Using the blockchain technology to improve the system security and transparency for digital coins transactions.

**Figure 1:**
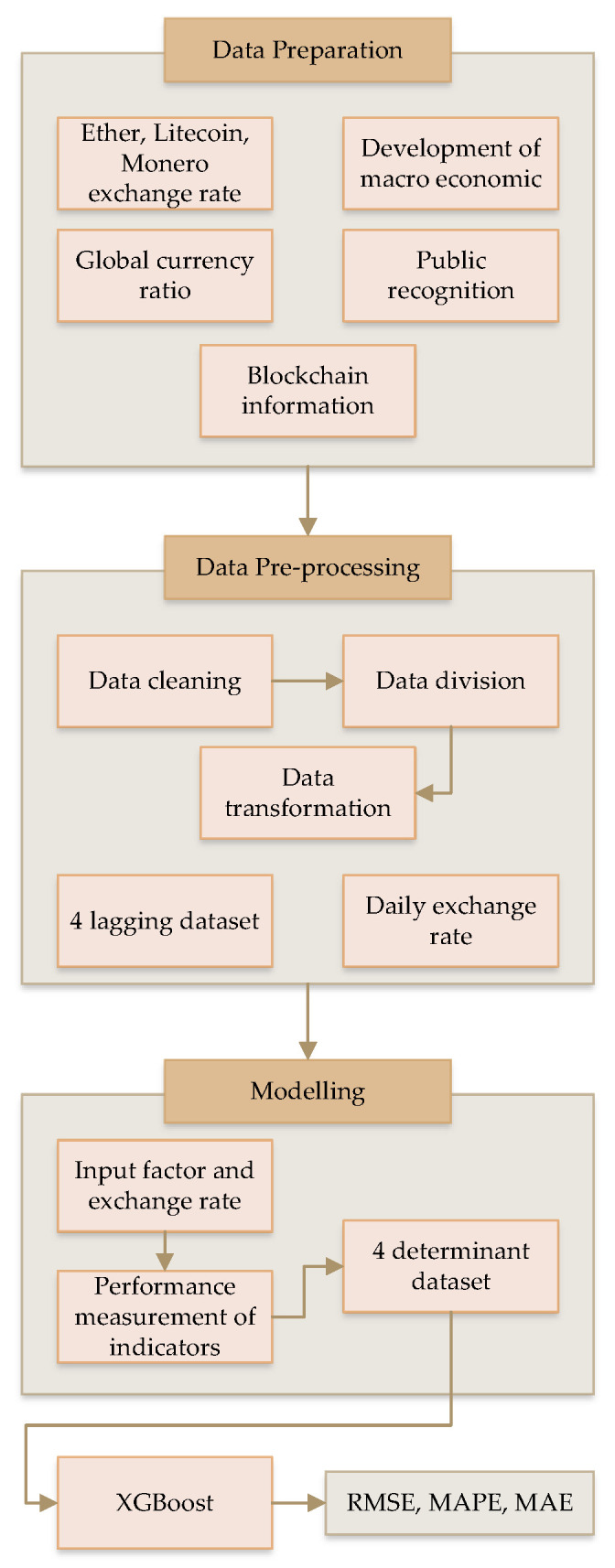
Overview of the employed model in this research.

The main reason for using XGBoost in the proposed framework is the performance of this algorithm. The main focus of this process is to improve the exchange rate of digital coins with higher performance and a more secure environment. Figure 1 presents the simple overview of the proposed exchange rate prediction process. There are three main steps in this process, namely, data preparation, data pre-processing, and modelling.

The rest of the process is divided as follows: Section 2 represents the brief literature review related to exchange rate prediction. Section 3 presents the proposed prediction process and blockchain structure for the digital coins exchange rate. Section 4 presents the implementation process and development environment details. We conclude this paper in the conclusion section.

## 2. Literature Review

In this section, a detailed explanation of the related research in this field is provided. The business operations in this world has become globalized in recent markets, and the economy has become decisive in every country so as to be successful on a stage of global purposes. Based on this situation, there is no way to ignore the currency.

### 2.1. Concept of Cryptocurrency

The popularity of cryptocurrency is based on the peer-to-peer network design, transaction cost, and ungoverned nature type [6,7]. These aspects cause increases in the of volume trading, the price of exchange, and volatility, which become key roles in media. There is a huge amount of recently developed studies, in terms of cryptocurrency for finance, covering, e.g., the efficiency of markets [8,9,10,11]. There are many systems related to Neural Networks (NN) for the cryptocurrency trading strategy [12,13,14,15]. Based on the various analyses, the neural network system uses the strategy of “buy and hold” in the process of bull trends, which causes the incompetence of information and produces unusual benefits [16]. Moreover, deep learning is proposed in many research works on recent technologies [17,18,19] for showing the price formulation for the behavior of trading. The first category of this topic in the state of the art is the Recurrent Neural Network (RNN), specifically, Long Short-Term-Memory (LSTM). Lahmiri et al. [20] prospected the utilization of LSTM and Generalized Regression Neural Networks (GRNN) in the prediction of prices for digital coins. Based on the process between these two algorithms, the LSTM has better performance than GRNN in terms of RMSE, based on the daily level. Tan et al. [21] compared the deep learning models with linear models of the financial market. ARIMA, Random Forest, Multi-Layer Perceptron, Prophet, and Regression models were tested during this process.

### 2.2. Prediction Models for Cryptocurrency

There have been many approaches, using machine learning algorithms, to the prediction of digital coins’ prices in recent years [22]. Sin et al. [23] predict Bitcoin’s price by applying the Linear Regression model (LR) and Support Vector Machine (SVM) regarding the time-series data information and daily-based approach for the 2012–2018 time period. The combination of various parameters applied in this prediction model is based on the lowest error rate. The applied filters are based on the different lengths of windows and different weights of coefficients. The price prediction of Bitcoin can accomplish the various length of windows with the usage of filters. Azari et al. [24] proposed the prediction model based on ARIMA to evaluate the future value of Bitcoin, regarding the available dataset from 2015 to 2018. The presented report of their results contains the minimum of 0.02 residual sum of squares. Hans et al. [25] compared the ARIMA model with Prophet by applying LSTM and multi-layer perceptron for cash flow prediction.

### 2.3. Concept of Knowledge Discovery for Cryptocurrency Technology

Knowledge discovery-distributed architecture demonstrates the incomplete knowledge of the local sites based on the merged information and distributed data [26,27,28]. This process happens between multiple nodes used to analyze the big data and the efficiency of the computation process [29]. The knowledge extraction uses multiple data mining and machine learning techniques to explain the data in possible details [30]. Mendis et al. [31] proposed the combined approach of blockchain and machine learning for privacy protection using blockchain nodes for training and aggregation.

Table 1 presents the comparison of recent existing work on cryptocurrency price prediction. There are six categories for this Table: type of digital coin, labels, transaction, features, applied algorithm, and performance.

## 3. Proposed Exchange Rate Prediction Approach

**Figure 2:**
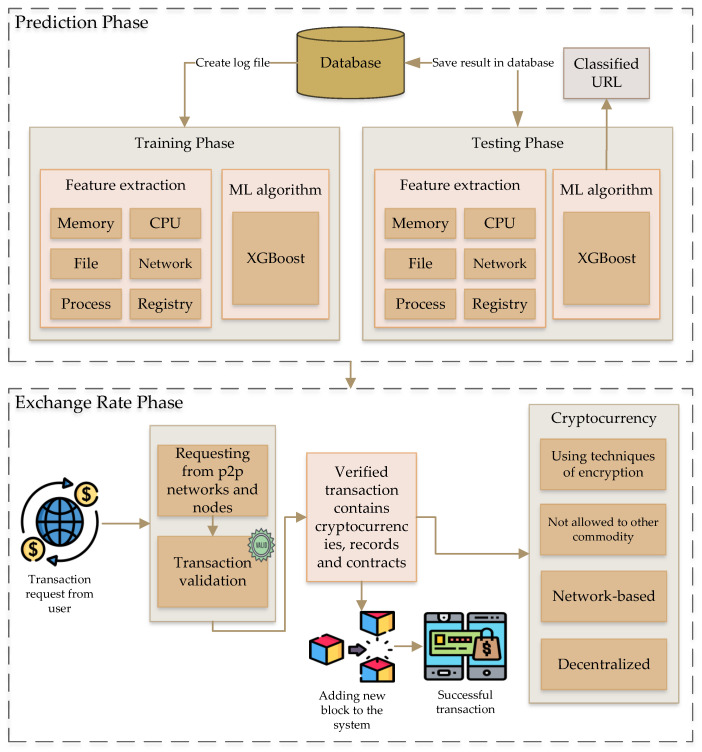
Overview of exchange rate prediction of the proposed system.

This section discusses two key processes: extreme gradient boosting-based exchange rate and blockchain-based exchange rate. The main approach applied in this system is to predict the digital coins’ price rate based on a combination of machine learning and blockchain frameworks to improve the performance of exchange more conveniently and securely. Figure 2 shows the overview of the proposed approach. In this process, we have applied the XGBoost machine learning model for the prediction of digital coins’ price. During this process, there are two main phases: the train phase and the test phase. The XGBoost algorithm contains the feature selection process and classification of URLs. The exchange rate prediction of the cryptocurrency is further processed in the blockchain network based on transaction validation and verification. The mentioned database in the prediction phase keeps the data related to the 30- and 90-day prediction of the transactions through XGBoost model.

### 3.1. Knowledge Discovery of the Cryptocurrecny Exchange Rate

Most of the time, the provided data does not give the enough information to the system for further processing. To do this, knowledge discovery is required, which, by applying various machine learning techniques, makes it possible to extract the knowledge from data for obtaining accurate results. One of the proposed system’s contributions is the knowledge discovery technique for predicting the cryptocurrency exchange rate. In this process, the main point is to use the knowledge discovery technique to extract the transnational information from the exchange rates by applying the XGBoost algorithm, which is described in detail below. The trans-national data information, which is used for knowledge discovery, is spread over the machine learning model for training, and this makes it possible to obtain data privacy in the knowledge discovery process. Another point of view of using this technique is to obtain clear information for the users who are doing the transactions, and their identity if the transactions are happening in the right direction. The knowledge discovery concept in this process is to extract the clear transactions information for improving the performance of the system’s security.

### 3.2. Extreme Gradient Boosting-Based Exchange Rate

XGBoost is a machine learning boosting algorithm famous for its high performance based on supervised learning. The highest usage of this algorithm is for the problem of classification and regression. This algorithm is preferred because of its high speed in core computation. The XGBoost working process is based on the following to predict the output. Equation (1) presents the prediction process. *E* is the dataset, *d* is the number of features, and *i* is the number of examples [46].

(1) $$E=\left(n_{x},d_{x}\right):x=1,...,i,n_{x}\in R^{y},d_{x}\in R$$

To predict the ${\hat{d}}_{x}$, the is process generated from Equation (2). *C* represents the total records of trees in the model. $c_{k}$ shows the last tree for this model.

(2) $$B_{x}=⌀\left(n_{x}\right)=\sum_{K=1}^{K}c_{k}\left(n_{x}\right),c_{k}\in C$$

Finding the best functions requires minimizing the loss and objective regularization by following Equation (3):

(3) $$Y\left(⌀\right)=\sum_{x}1\left(d_{x},B_{x}\right)+\sum_{K}\Omega \left(c_{k}\right)$$

*Y* is showing the loss function based on the differences of prediction between the output value ${\hat{d}}_{x}$ and actual value $d_{x}$. Ω presents the model complexity and avoids over-fitting. This process evaluates by following the Equation (4):

(4) $$\Omega \left(c_{k}\right)=\gamma L+\frac{1}{2}{\lambda ||V||}^{2}$$

*L* presents the total records of leaves in the tree, and *V* shows the weight of every leaf.

### 3.3. Blockchain-Based Exchange Rate

**Figure 3:**
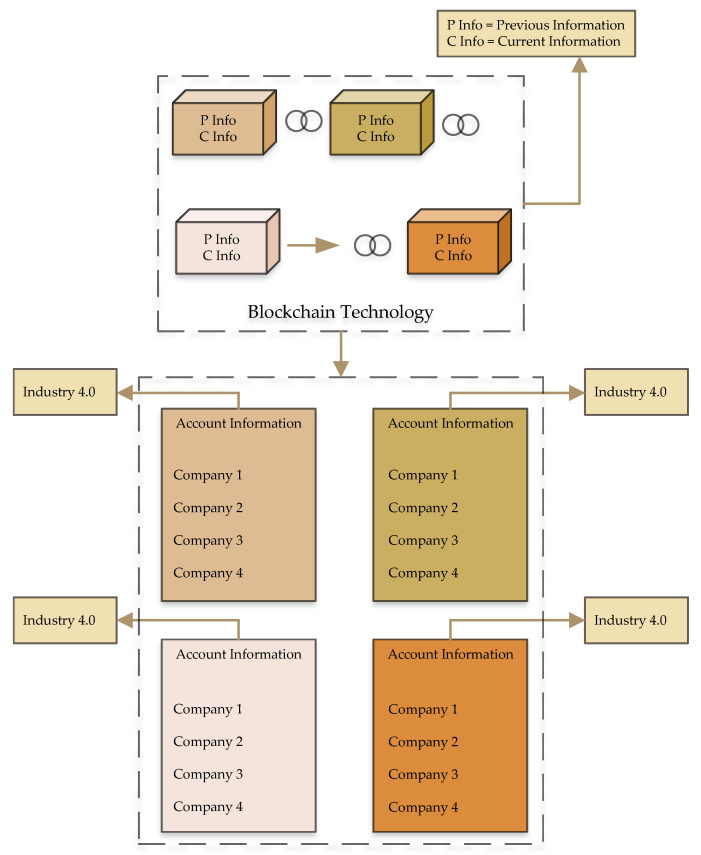
Overview of exchange rate based on the blockchain framework.

Modeling a complex system in a blockchain framework is based on a network, and it is an essential perspective of this process. The network is available everywhere in social-, physical-, and technical-based interconnected components and economic systems. In the past 20 years, the study of complex networks presents the property structures of real-world networks, such as small-world phenomena, scale-free properties, and the mechanism of similar network formation. The network analysis for the cryptocurrency transaction study improves specifically in terms of the characteristics of user activities and checking the network structure and temporal properties. As mentioned previously, Bitcoin is the first cryptocurrency created for lots of media coverage. By this time, many other cryptocurrencies have also emerged in the world of the digital coin. There are two key bases for cryptocurrency, which are decentralized networks and computer cryptology. Cryptology authorizes the trans-national data security and saves them into the blockchain, a public ledger. Across the network, the ledger distributes the nodes, and the computational power contributes to the encryption of transactions and cross-validation. The cryptocurrency can process without any limitations from a central authority. Figure 3 shows the exchange rate process in terms of the blockchain framework. The cryptocurrency uses a blockchain framework, the money ledger in the advanced level, and avoids the double-spending issue without the requirement of trust authority based on the central server.

**Figure 4:**
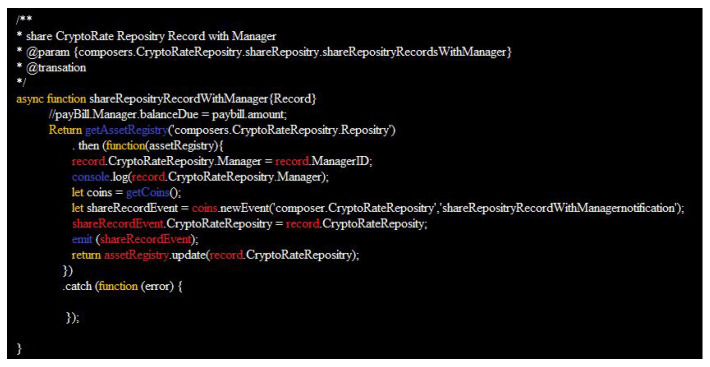
Transaction process function based on the blockchain framework.

Blockchain security features define, based on user ledgers, chains of blocked and decentralized applications. The ledger has the responsibility of recording every transaction’s information in the blockchain. The ledger information is changeless and famous for decentralized applications. In this case, no one can gain access to the data information or its read-only file for users. Each block contains the hash value, and blocks are connected to each other based on previous hash information. In case of attempts to change the data information, the hash will change, and it effects the entire chain. This increases the protection of sensitive data information. Similarly, the blockchain approach is a peer-to-peer communication that contains the network nodes, and all these thousands of nodes have to contain the copy of the distributed ledger. This process contains the transaction authentication. In this case, if the node does not allow the transaction, then further processes cannot happen. This process avoids fraudulent transactions in the network. Figure 4 shows the process of cryptocurrency exchange rate in the blockchain framework. Cryptocurrency pricing based on the blockchain network follows the conditions of marketing. This aspects assumes the value of the digital currency, traditional currency, and exchangeable currencies, which are under the control of a central bank. The cryptocurrency supply is evaluated following Equation (5) to estimate the Ether exchange rate. *F* is the capacity of total cryptocurrency circulation.

(5) $$S_{F}=P_{F}F$$

The cryptocurrency demand *P* is evaluated based on the following Equation (6). *W* is the cryptocurrency velocity, *G* is the economy size, and *P* is the price level.

(6) $$I_{F}=\frac{P_{G}}{W}$$

## 4. Experimental Results

In this section, the results of the exchange rate prediction approach are explained in detail. We have described the development environment, prediction process details, and the performance evaluation of the proposed system.

### 4.1. Development Environment

Table 2 presents the development environment of the proposed exchange rate prediction method. There are two key points used: the combination of machine learning and blockchain. The machine learning section contains Windows 10 as an operating system, IE, Firefox, and Chrome as a browser, Python and IDE as a programming language, and the machine learning technique is XGBoost.

### 4.2. Processing and Dataset

There is lots of information related to cryptocurrency exchange rate on social media, but not all are true. The knowledge extraction in this process helps to analyze the information based on their trustworthiness and a comparison with the recent achievements in this field. The https://digitalcoinprice.com/ website is one of the complete websites related to giving detailed information on price changes and the procedings of exchanges based on the date and time, which is the best option for exchange rate prediction. Every element gives the information related to the sender, an ID of exchange, incentives, and a timestamp. It contains different lines of various collectors regarding the same ID of exchange. This information is helpful for rearranging the collected list based on the client material by pointing the calculation of union-find of random type exchange, which considers only a single element without the need of having various accounts for exchange. Data representations give the ability to explore different properties for the prediction based on various features. The data which are applied for this process is from 2018 to 2021, and the process of prediction is for 7, 30, and 90 days. We have used 80% of the data for training and 20% of the data for testing. After the price extraction of cryptocurrency, the details are saved into JSON files and converted into CSV for comfortable analysis. The data are partly taken from the above-mentioned website, which is an open-source file, and some are taken from the available projects which focus on this field, which are not open-source.

### 4.3. Performance Evaluation

The process of the experiments and simulation is based on the available data in Ether, Litecoin, and Monero explorer with the details of daily transactions. Then, the collected data are analyzed the parameters are pre-processed based on the date, highest price, lowest price, whether they are open or closed, and the quote volume, and are average weighted. Nomenclature shows the details of the notations used in this process.

The performance evaluation in this system was evaluated regarding the MAE, RMSE, and MAPE. The following Equations (7)–(9) present the details [47].

(7) $$\mathrm{MAE}=\frac{1}{m}\sum_{x=1}^{m}\left|z_{x}-{\hat{z}}_{x}\right|$$

(8) $$\mathrm{RMSE}=\sqrt{\frac{1}{m}\sum_{x=1}^{m}{\left|z_{x}-{\hat{z}}_{x}\right|}^{2}}$$

(9) $$\mathrm{MAPE}=\frac{100}{m}\sum_{x=1}^{m}\frac{|z_{x}-{\hat{z}}_{x}|}{z_{x}}$$

Table 3 gives the details of the statistical analysis of the XGBoost algorithm with the other state-of-the-art algorithms, namely, CNN, Arima, MLP, and LSTM.

#### Feature Extraction

An important part of data pre-processing is feature extraction, which is necessary for improving the performance of the proposed model. The extracted features are based on applying various approaches. First of all, the importance of features is specified based on the XGBoost algorithm. Next, the number of features is decreased by checking for cross-correlation and multi-collinearity. To do this, the Pearson correlation and variance inflation factor were applied. The subset result of features contains low correlation and important high values without multi-collinearity. This process continues for all three intervals: 7 days, 30 days, and 90 days. When the prediction for the *m*th day is reached, the process of feature selection creates a new feature subset, which is the best option for the interested period, e.g., the features for predicting the 7-day exchange rate cannot predict the price of the coming 90th day.

**Figure 5:**
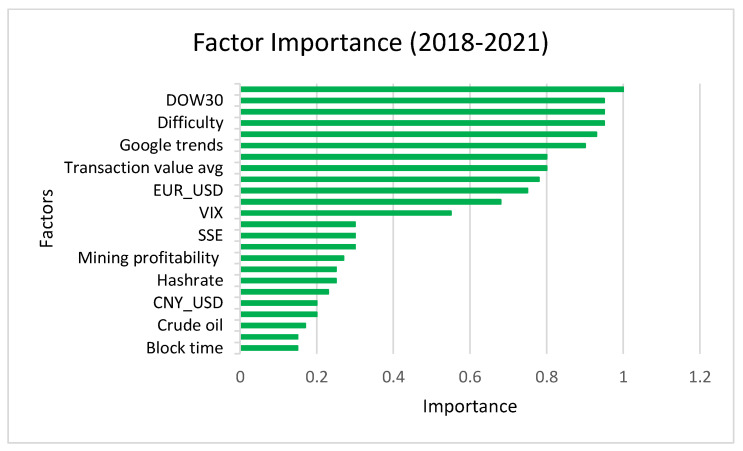
Different factors’ importance from 2018 to 2021.

Suitable variable selection for the input of the process is difficult but necessary for ML algorithms to provide good performance. The input variables’ selection ensures that the defined model can extract the relationship of the target value and input it in the training process. The input variables are the exchange ID, timestamp, sender information, and incentives. In the presented exchange rate system, cross-correlation is applied for evaluating the similarity between variables and the delayed value. Figure 5 shows the feature importance of the presented exchange rate of digital coins. As mentioned, the XGBoost algorithm was applied for this process’s feature selection to evaluate the importance of factors. The factors are in rows based on the most important one. This shows the result of predictable technological factors’ identification. To do this, the changes of exchange rate are considered in different periods, as mentioned, from 2018 to 2021. The factor importance considers difficulty, Google trends, hashrate, block time, etc.

Table 4 gives the detail information related the technical indicators of the raw features.

### 4.4. Prediction

Table 5 shows the result of the Ether, Litecoin, and Monero cryptocurrencies statistic prediction accuracy. The baseline results present that the prediction based on the classifier gives the repeated class for every currency most of the time. The table shows the records of Mean, Median, Var, Min, and Max.

Table 6 presents the results of the ten-day prediction based on the XGBoost algorithm for digital coins exchange rate. There are four columns to show the date, the actual rate of coins, the predicted rate, and the error rate.

The error rate is evaluated based on the following Equation (10):

(10) $$Error.rate\left(\%\right)=\frac{Actual-Predicted}{Actual}*100$$

Table 7 presents the cryptocurrencies’ breakdown pattern classes. As is shown in the Table, there are two classes, 0 and 1. Class 1 shows the valuing based on USD, and class 0 is for the rest.

**Figure 6:**
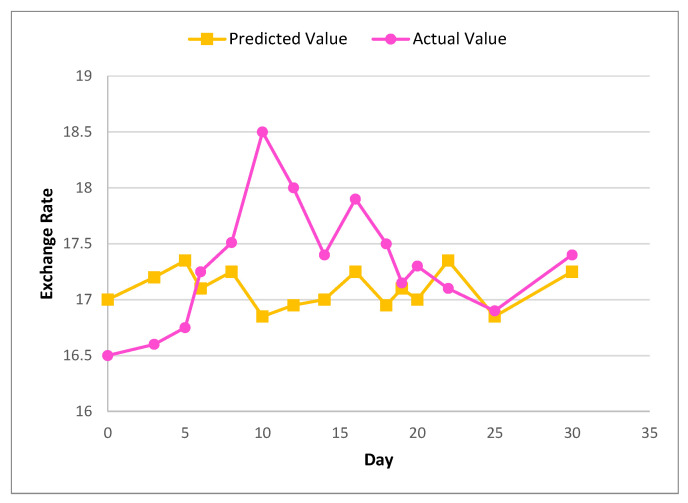
Actual and predicted value of Ether using XGBoost for 30 days.

**Figure 7:**
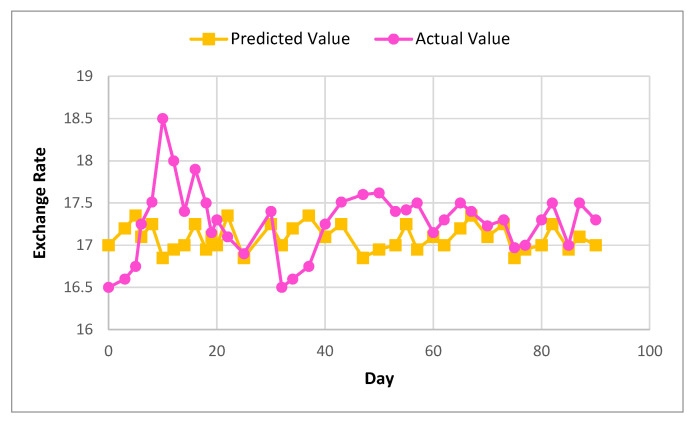
Actual and predicted value of Ether using XGBoost for 90 days.

Figure 6 and Figure 7 show the actual and predicted value differences of Ether, using XGBoost for exchange rate prediction for 30 and 90 days.

**Figure 8:**
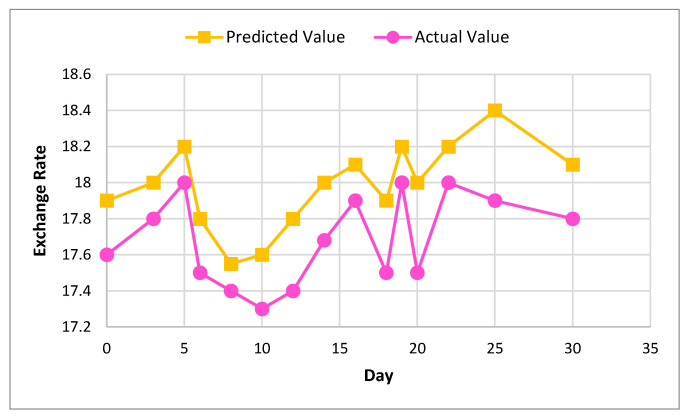
Actual and predicted value of Litecoin using XGBoost for 30 days.

**Figure 9:**
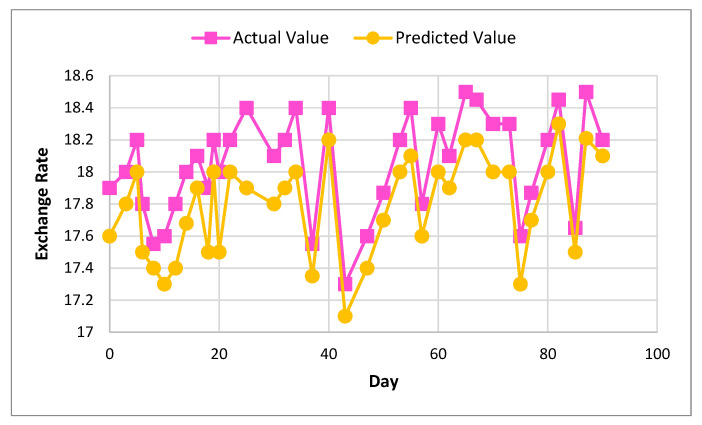
Actual and predicted value of Litecoin using XGBoost for 90 days.

Figure 8 and Figure 9 show the actual and predicted value of Litecoin, using XGBoost for exchange rate prediction for 30 and 90 days.

**Figure 10:**
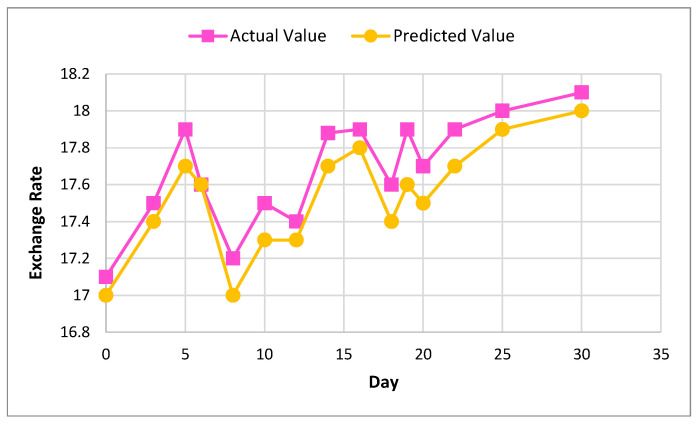
Actual and predicted value of Monero using XGBoost for 30 days.

**Figure 11:**
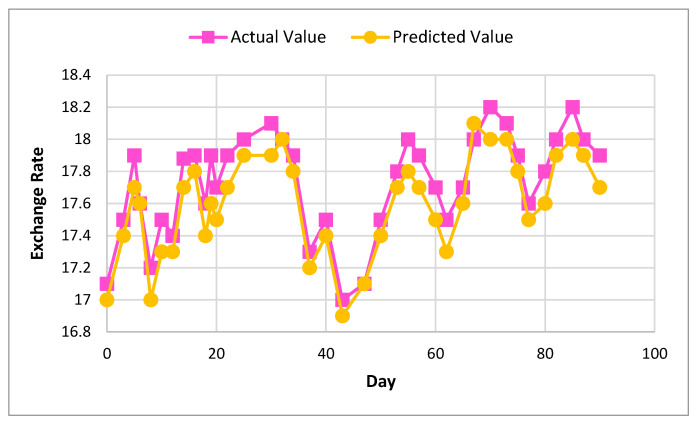
Actual and predicted value of Monero using XGBoost for 90 days.

Figure 10 and Figure 11 show the actual and predicted value of exchange rate prediction of Monero for 30 and 90 days.

**Figure 12:**
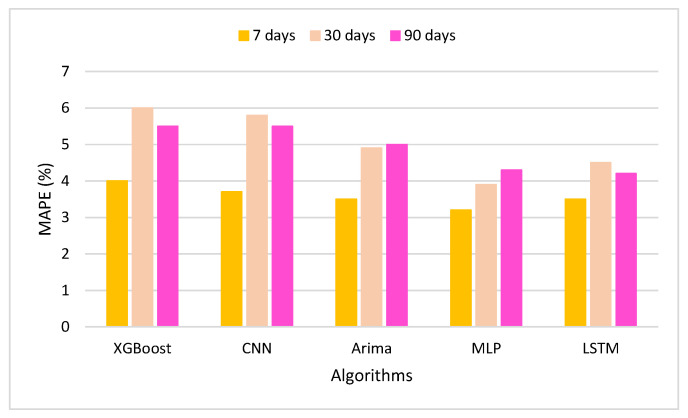
MAPE of the classification model.

Figure 12 shows the machine learning algorithms’ performance in 7 days, 30 days, and 90 days, based on MAPE. The comparison of XGBoost’s performance is with CNN [48], MLP [49], and LSTM [50], as is shown in the Figure. MAPE evaluates the average error of the actual values over time and changes. This assumes the preference between days that shows better prediction. The LSTM approach is a promising deep learning approach, which appears to perform well for analyzing the numerical dataset. LSTM can not handle the data in case of different sizes of the input and output values [51].

**Figure 13:**
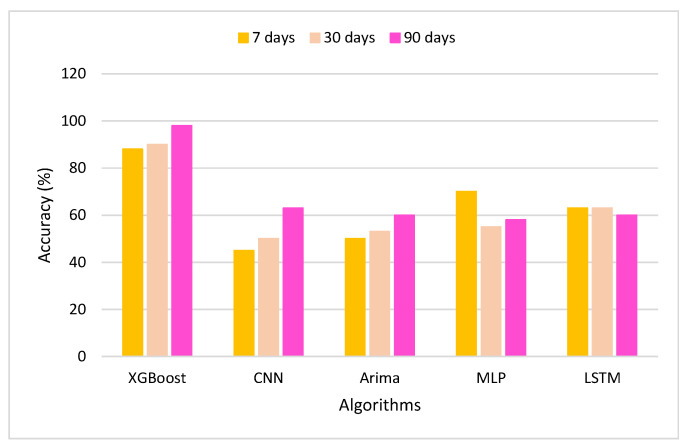
Accuracy of the classification model.

Figure 13 shows the machine learning algorithms’ accurate records that are compared in the proposed approach. As shown, the applied XGBoost algorithm has the highest accuracy score during the 7-day, 30-day, and 90-day periods. The CNN algorithm achieves the lowest score during this process.

## 5. Conclusions

Digital coins exchange rate prediction can provide the traders of cryptocurrency and stock brokers an upper hand in the market. This algorithm provides an accurate result, which makes the trained model deployed. Compared with other algorithms, XGBoost has great results in the exchange rate prediction of the daily records of digital coins, namely, Ether, Litecoin, and Monero. The performance of the prediction model was evaluated based on MAE, RMSE, and MAPE. In addition, the XGBoost model contains a smaller record of RMSE as compared to others. The research finding of this approach focuses on the daily exchange rate prediction of digital coins by utilizing various resources and providing a new approach in this area. The knowledge discovery in this process focuses on the incomplete information of the cryptocurrencies in social media and analyzes the cryptocurrency data to obtain better outputs for further processing. The blockchain improves the security and transparency of the following exchange rate approach, providing trust and acceptable assessments to users in the coin market.

## Figures and Tables

**Table 1:** Comparison of recent price prediction tasks of cryptocurrency.

Author	Cryptocurrency	Labels	Transaction	Features	Applied Algorithm	Performance
1 [32]	Bitcoin	Address exchange, Service of gambling	Nov 2018	Embedding	HDDT+ ECOC	0.91%
2 [33]	Bitcoin	Entities of exchange, Entities of gambling, Entities of mining pool, Entities of market place	0 to 561 blocks	Network, Volume	GBDT, Cascading	0.99%
3 [34]	Bitcoin	Addresses of mining pool, Minors, Mixing service, Exchange	520.850 to 520.950 blocks	Embedding, Temporal, Volume	RF	0.96%
4 [35]	Bitcoin	Faucet offering, Exchange, Gambling, HYIP	2009–2017	Network, Volume, Temporal	RF	0.70%
5 [36]	Bitcoin	Address of exchange, Faucet, HYIP, Mining pool	2009–2018	Network, Volume, Temporal	Light GBM	0.86%
6 [37]	Bitcoin	Exchange entities, Mining pool, Darknet market place	0–514.971	Network, Volume	Temporal	0.91%
7 [38]	Bitcoin	Entities of exchange, Hosted wallet, Darknet market place, Service of merchant	Not disclosed	Network, Volume, Temporal	Extra trees	96%
8 [39]	Ethereum	Authors of smart contract	-	Stylometrics	RF	91%
9 [40]	Litecoin	Daily price	2009–2018	Market information, Network	RF	Prediction contribution with motif feature
10 [41]	Ethereum	Daily price	2016–2018	Difficulty of mining, Volume, Market information	LR	0.99%
11 [42]	Litecoin	Daily price	2017–2019	Difficulty of mining, Volume, Market information	SNN	Lowest MAPE
12 [23]	Bitcoin	Daily Price	2015–2017	Difficulty of mining, Market information, Volume	GASEN	64%
13 [43]	Bitcoin	5 min price direction in one day	2017–2019	Difficulty of mining, Market information, Volume	LR, LSTM	66%
14 [44]	Bitcoin	30th, 90th, and next- day price direction	2013–2019	Difficulty of mining, Market information, Volume	LSTM	MAE, RMSE, MAPE, 62% to 65%
15 [45]	Bitcoin	Direction and daily price	2017	Network, Volume	PDE	0.82%

**Table 2:** Development Environment.

Name	Components	Description
Machine Learning	Operating System	Windows 10
	Browser	IE, Firefox, Chrome
	Programming Language	Python, IDE
	ML Algorithm	XGBoost
Blockchain Network	Operating System	Ubuntu Linux 1804 LTS
	Programming Language	Node.js
	CPU	Intel(R) Core(TM) i7-8700 @3.20 GHz
	Docker Engine	V18.06.1-ce
	Docker Composer	V1.13.0
	IDE	Composer Playground
	Memory	12 GB

**Table 3:** Statistical testing comparison.

Algorithm	MAE	RMSE	MAPE
XGBoost	0.608	0.765	0.005
CNN	1.720	2.188	0.014
Arima	0.1748	2.6812	0.0190
MLP	0.1748	0.2621	0.0014
LSTM	0.083	0.3091	0.0007

**Table 4:** Technical indicators’ raw features.

Features	Description
Block Size	Transaction information
Transaction	Payment records which are sent and received
Difficulty	Average of daily mining difficulty based on the number of blocks
Sent Records	Distinct digital coin addresses based on daily payment records
Average Transaction Value	Digital coins’ transactional average value
Mining Profitability	Every terahash profit per day based on USD
Reward Ratio Fee	The transactions sent ratio for reward verification based on user transaction records
Median Transaction Fee	Digital coins’ median transaction
Average Transaction Fee	The received transaction fee from minor for verification
Block Time	Required time for block mining
Median Transaction Value	Digital coins’ median transaction value
Hashrate	Digital coins’ daily computational capacity
Active Addresses	The participating addresses in the transaction

**Table 5:** Statistic prediction accuracy records of cryptocurrency.

#		Mean	Median	Var	Min	Max
Ether	XGBoost	0.6988	0.7011	<0.002	0.6963	0.7049
	CNN	0.6898	0.6898	<0.002	0.6849	0.6966
	Arima	0.676	0.6776	<0.002	0.6754	0.6950
	MLP	0.6623	0.6623	<0.002	0.6539	0.6625
	LSTM	0.6389	0.6587	<0.002	0.6339	0.6625
	Baseline	0.6578				
Litecoin	XGBoost	0.7874	0.7879	<0.002	0.7835	0.7916
	CNN	0.7676	0.7674	<0.002	0.7616	0.7717
	Arima	0.7598	0.7587	<0.002	0.7415	0.7518
	MLP	0.7195	0.7197	<0.002	0.7158	0.7235
	LSTM	0.7198	0.7194	<0.002	0.7079	0.7248
	Baseline	0.7199				
Monero	XGBoost	0.8995	0.8995	<0.002	0.8967	0.9139
	CNN	0.8698	0.8611	<0.002	0.8649	0.8744
	Arima	0.8650	0.8644	<0.002	0.8632	0.8720
	MLP	0.8594	0.8591	<0.002	0.8659	0.8558
	LSTM	0.8585	0.8587	<0.002	0.8614	0.8562
	Baseline	0.8579				

**Table 6:** Prediction results of ten-day exchange rate changes.

Date	Actual Rate	Predicted Rate	Error (%)
1 Feb 2021	14,942	14,920.74	0.10
2 Feb 2021	14,974	14,915.26	0.42
3 Feb 2021	14,952	14,881.75	0.40
4 Feb 2021	14,952	14,881.75	0.40
5 Feb 2021	14,952	14,881.75	0.40
6 Feb 2021	14,992	14,998.68	0.78
7 Feb 2021	14,960	14,998.18	0.56
8 Feb 2021	14,975	14,985.69	0.76
9 Feb 2021	14,892	14,874.19	0.31
10 Feb 2021	14,954	14,962.69	0.07

**Table 7:** Three cryptocurrency breakdown pattern records.

#		Ether	Litecoin	Monero
Train	0 1	65.79% 56.43%	71.58% 49.64%	85.55% 36.67%
Test	0 1	65.78% 56.44%	71.99% 49.23%	85.79% 36.42%

## Data Availability

Not applicable.

## Nomenclature

$z_{x}$	Model output
$\hat{Z}$	Target Value
m	Time variable
$\hat{A}$	First variable mean
$\hat{B}$	Second variable mean
A	Ideas about the shared contents
B	Second variable
E	Data representation
d	Number of features
i	Number of examples
$c_{k}$	Last tree
C	Total record of trees
Ω	Model complexity
Y	Model loss function
L	Total leaves records
V	Weight
F	Cryptocurrency circulation
W	Velocity
I	Cryptocurrency demand
G	Economy size
P	Price level
